# Purification of chemically fixed HIV-1 spikes for oriented display on nanoparticles

**DOI:** 10.1186/1742-4690-9-S2-P347

**Published:** 2012-09-13

**Authors:** MB Zwick, D Leaman

**Affiliations:** 1 The Scripps Research Institute, La Jolla, CA, USA

## Background

HIV-1 neutralizing antibodies (Abs) bind to the envelope glycoprotein (Env) spike, which functions as a trimer of gp120-gp41 heterodimers that is anchored in the viral membrane. However, Env trimers are low in copy number and coexist with irrelevant forms of Env and its byproducts, which typically elicit non-neutralizing Abs.

## Methods

Here, we have attempted to generate immunogens by fixing trimeric spikes of HIV-1JR-FL using a defined chemical crosslinker, purifying the crosslinked spikes from virions, and immunodepleting them of irrelevant Env contaminants using non-neutralizing Abs.

## Results

Purified, crosslinked spikes were bound by a panel of neutralizing Abs, but not by non-neutralizing Abs, and are virtually devoid of non-trimeric Env. However, at least one neutralizing epitope on gp120, the crown of V3, appears to be occluded by crosslinking. An immunization was performed using the purified, crosslinked Env trimers as a boosting agent following a DNA prime using full-length env, either as soluble protein or captured onto small proteoliposome nanoparticles (PLNs). The binding titers of the Ab response to crosslinked Env spikes were quite weak, possibly reflecting an overall weaker immune response. Boosting animals with trimer-PLNs however elicited a qualitatively different neutralizing Ab response than uncrosslinked Env on virions, with sporadic activity against neutralization-resistant HIV-1 isolate JR-CSF, reduced neutralization of sensitive Tier 1 isolates, and reduced antibody responses against host protein.

## Conclusion

Immunizing animals using purified, crosslinked Env spikes captured on PLNs elicited a qualitatively different and broader neutralizing Ab response than uncrosslinked, heterogeneous Env. We conclude that with further changes to the crosslinking and immunization strategy crosslinked and purified Env spikes hold promise as a vaccine candidate.

